# Atropia, Duboisia, Eserine, Pilocarpine and Muscarine

**Published:** 1879-04

**Authors:** E. L. Holmes

**Affiliations:** Chicago


### ARTICLE III.

### ATROPIA, DUBOISIA, ESERINE, PILOCARPIN AND MUSCARINE.

By Dr. E. L. HOLMES, of Chicago. Read before the Chicago Medical Society, March 17, 1879.

A few facts regarding the last four alkaloids, as used in ophthalmic practice, will not, I trust, be without interest to members of this society.

The action of atropia is so well known to the profession that it is necessary simply to allude to it in this paper to compare its effects with those of the other agents above mentioned; especially as a recent paper, read before this society, discussed those injurious effects of atropine upon the eye, which are not very unfrequently observed by every specialist.

Although the addition of sodæ sulph. or of morph. sulph. to the solution of atropine reduces its tendency to irritate the eye, a new mydriatic has been thought desirable.

Hyoscyamin and extracts of hyoscyamus, as well as those of stramonium, are unreliable.

De Wecker, of Paris, was one of the first to bring to notice a new remedy, duboisia, obtained first (March, 1878) by Petit, of Paris, and soon after by Garrold, of London. This is a product of a small tree growing in Southern Australia.

I have seen but two specimens of the drug; a single grain of duboisia, which is a yellowish resinous substance, and a gram of the neutral sulphate of duboisia, a light brown syrup-like fluid—price, $15. If experience shall demonstrate that this remedy is so valuable as some have predicted, its cost will undoubtedly be decreased. A dollar a grain, however, is a small price for a remedy which alone, in an exceptional case, may be able to save an eye.

According to De Wecker and others, who have used duboisia in cases in which atropine was not tolerated, it possesses the properties of dilating the pupil and paralyzing the accommodation more rapidly than atropine.

I have substituted duboisia for atropine in a case of keratitis, in which prolonged use of the latter had caused great irritation of the conjunctiva and swelling of the lids. These symptoms at once subsided with the use of duboisia, 0.06, aqua, 60.0.

Two valuable remedies have in recent times been added to ophthalmic materia medica—eserine, an active principle of Calaber bean, and pilocarpine, derived from jaborandi.

Both alkaloids possess the property of causing contraction of the pupil and spasm of the ciliary muscles. Patients under its influence see distinctly objects only relatively near. Patients under the influence of atropine see distinctly only objects relatively far.

The influence of eserine and pilocarpine is much less durable than that of atropine.

As far as I am aware, experiments with eserine have been more numerous than those with pilocarpine, although it has been stated that the nitrate of pilocarpine, as also the muriate, which is most in use, is less irritating to the tissues of the eye than eserine.

The solution of eserine loses its strength quite rapidly by decomposition, while that of pilocarpine is stable and, I think, less expensive than that of eserine.

Eserine has been used with benefit in the following conditions: In glaucoma, staphyloma and conical cornea, ulcer of the cornea, with or without hypopion; when the iris has been recently engaged in a penetrating wound or perforating ulcer of the cornea. It is also stated that it reduces suppuration of the conjunctiva (keratitis), and inflammation of the nasal duct and of the sack. While atropine is supposed to dilate the vessels of the eye, eserine contracts them.

These alkaloids are both contra-indicated when there is a tendency to iritis. They are of almost absolutely no benefit in producing a permanent contraction of the pupil in idiopathic mydriasis.

I need but refer to the fact that while atropine may relieve excessive sweating, pilocarpine induces it.

I have prescribed nitrate of pilocarpine in two cases of keratitis punctata, which is very often an obstinate disease. It not only produced no irritation but gave perfect and speedy relief to the patients.

In a case of staphyloma with an increase of tension, and in a conical cornea with ulceration and conjunctivitis, but with no increase of tension, pilocarpine relieved the active symptoms. Its use in three cases of ulcer of the cornea, with quite severe conjunctivitis, was without benefit. It was applied to the conjunctiva in a one per cent. solution.

*Muscarine* is one of the active principles of poisonous mushrooms. It has been but little used in treating diseases of the eye. Its singular properties, however, have led physiologists to study its action, not only on the general system, but also on the eye. It is described as a syrup-like alkaloid, soluable in water, without color, taste or smell. Experiments show that its effects are quite constant in causing spasm of the accommodative apparatus.

Its power to contract the pupil varies in degree somewhat in different individuals.

By suitable union of atropine and muscarine, there may be dilatation of the pupil and spasm of the ciliary muscle in the same eye.

Muscarine in poisonous doses produces an arrest of the heart's action by its effects on the inhibitory nerves of this organ. Atropine, administered internally, or especially hypodermically, tends to neutralize the effects of muscarine on the heart.

It may be of interest to the general practitioner that atropine in doses of 1-100 of a grain, given at suitable intervals, should be administered to patients who have partaken too freely of poisonous mushrooms.

The literature of this subject is confined principally to the *Archives of Ophthalmology—Monatsblaetter f. Augenheilkunde* and the *Journal of Mental and Nervous Diseases*, which give references to the labors of various experimenters in France and Germany.

